# An Autopsy Case of Misdiagnosed Wernicke's Syndrome after Intragastric Balloon Therapy

**DOI:** 10.1155/2018/1510850

**Published:** 2018-02-13

**Authors:** Paola Vellante, Aldo Carnevale, Cristian D'Ovidio

**Affiliations:** Department of Medicine and Aging Sciences, Università degli Studi “G. d'Annunzio”, Via Dei Vestini, 66100 Chieti, Italy

## Abstract

Intragastric balloon (IGB) therapy is a widely used technique to counter obesity, and it is known to be safe and effective. Although there can be a high rate of side effects following IGB therapy, most are self-healing and they are mainly accommodative in nature. Few cases of Wernicke's syndrome under IGB therapy have been described in the literature, and to the best of our knowledge, none have been fatal. We present here a case of a 51-year-old woman who underwent IGB therapy over 8 months. Late diagnosed Wernicke's syndrome that first appeared as lower limb neuropathy progressively immobilized the patient, until she required bed rest. Finally, a major complication of pneumonia caused her death. Wernicke's syndrome has been mainly related to bariatric surgery techniques, but it must also be linked to IGB therapy (and also to other restrictive weight-loss interventions). As the use of IGB therapy spreads, the literature must alert physicians to this complication. Wernicke's syndrome is a severe but reversible condition when diagnosed and treated early.

## 1. Introduction

Obesity is a growing health problem, and once lifestyle modifications and pharmacotherapies have failed, intragastric balloon (IGB) therapy represents a safe and effective treatment that can be considered before bariatric surgery techniques. IGB therapy is indicated for patients whose body mass index (BMI) is from 30 kg/m^2^ to 40 kg/m^2^, to prepare these superobese patients for bariatric surgery. It also represents a temporary treatment of choice. Nowadays, thousands of IGBs are placed globally, due to their versatility, low invasiveness, and safety.

Intragastric balloon placement is a minimally invasive procedure that was first performed in the early 1980s, and it has become popular in Europe only in recent years [1]. The use of IGB therapy has spread easily, such that the US Food and Drug Administration is going to approve more types of IGBs [2]. Of note, to correctly manage IGB therapy, an interdisciplinary approach is recommended, with the patients enrolled in a medically supervised weight-loss program.

There have been few reported cases of Wernicke's syndrome related to IGB therapy [3, 4]. In these cases, the diagnosis of Wernicke's syndrome was made in its early stages and was immediately treated. In contrast, there are no reports of late Wernicke's syndrome diagnosis (i.e., after IGB therapy) in the international literature.

The aim of this case report is to draw attention to the possible onset of Wernicke's syndrome during IGB therapy. Patients should be informed that Wernicke's syndrome can be a side effect of IGB therapy.

## 2. Case Report

We present here an autopsy case of a 51-year-old woman who underwent IGB therapy. At the time of the IGB placement, her BMI was 46.48 kg/m^2^. Six months later, her BMI had decreased to 30.5 kg/m^2^. Due to the lack of follow-up reports, it is unknown whether the patient missed any control visits or whether she was adequately followed up.

Seven months after the IGB placement, the patient arrived at the Emergency Unit complaining of fever, cough, lower limb asthenia, and general malaise. She was diagnosed with bronchial pneumonitis, and antibiotic therapy was prescribed. The patient was then discharged and sent to be followed up by her general practitioner. The IGB was removed 1 month later (i.e., it remained in situ for 8 months since its placement).

After a week, the patient was admitted to the hospital for asthenia, memory deficit, and progressive worsening of her general malaise. During this hospitalization, her relatives reported a history of vomiting that was correlated with food intake and that had started soon after the IGB placement. It then appeared to worsen from the fourth month after the IGB placement. Moreover, the relatives reported a diagnosis of relatively unspecified peripheral neuropathy.

The patient then contracted severe nosocomial pneumonia, and she soon progressed into septic shock. After an episode of cardiac arrest, the patient was transferred to the Intensive Care Unit, while in a coma.

The laboratory examinations during the hospitalization of this patient revealed dysproteinemia, with total protein of 4.6 g/dL (normal range: 6.6–8.7 g/dL) and albumin of 21.4 g/L (normal range: 35–52 g/L). Her serum thiamine was never determined, although thiamine was administered since the date of her cardiac arrest. After this episode, cerebral computed tomography showed hypodensity, edema, and vascular problems in the cerebellum vermian and encephalic trunk, and on the basis of the patient history, a consultant neurologist hypothesized Wernicke's syndrome at the origin of her illness. Eight days later, the patient was declared brain death.

The Prosecutor's Office of Pescara (Italy) called the Institute of Legal Medicine of Chieti to perform an autopsy. The autopsy revealed bilateral pneumonia, submucosal congestion in the gastric antrum, leucocyte infiltration into both the encephalic trunk and the mesencephalon, hemorrhagic extravasation, and multiple inflammatory infiltration in the cerebellum, which has hindered certain histological diagnosis of Wernicke's syndrome.

The following fatal sequence can be hypothesized. The IGB therapy appears to have caused persistent vomiting, which was generally ignored by the patient herself possibly due to her not well defined eating disorder with no specific cause. However, there are no comprehensive medical reports available. As the neurologist hypothesized, these events appear to have led to thiamine deficiency, which appeared initially as neuropathy and progressive immobility and then as Wernicke's syndrome. The immobility of the patient had a major role in her impaired pulmonary ventilation, which led to serious pneumonia. Finally, cardiac arrest while in septic shock caused a pontocerebellar infarction and brain death.

## 3. Discussion

The mechanism by which IGB induces weight loss is not fully understood [5]. It is believed that IGB reduces the gastric reservoir, leading to early and prolonged satiety and delayed gastric emptying. This would stimulate the gastric baroreceptors and also regulate hormone-mediated signal transduction [6]. On the contrary, IGBs would have no direct effects on nutrient absorption and metabolism.

Generally, the IGB is removed after 6 months, regardless of the BMI reached by the patient [7]. In cases of intolerance, the IGB can be removed earlier [8]. However, prolonged IGB therapy up to 14 months has been reported to maintain satiety and improve weight loss, without any safety issues [9].

In the present case, the IGB therapy duration of 8 months cannot be considered as the direct and only cause of Wernicke's syndrome, due to the lack of follow-up data. According to the literature data, persistent vomiting can be considered as the main cause of Wernicke's syndrome in this case. Nevertheless, Wernicke's syndrome has rarely been related to IGB therapy [3, 4].

The more common side effects of IGB therapy occur at relatively high rates, but most of these are self-limiting [6]. They are mainly accommodative in nature (i.e., nausea, abdominal pain, vomiting, and dyspepsia), and they generally decrease in frequency over time. Severe acid reflux can exacerbate the situation in ≤7% of patients. Major complications during IGB therapy occur in ≤2% of patients, and these include gastrointestinal ulceration, dehydration, luminal obstruction, and perforation [10]. There are also reports of fatal complications following IGB therapy, although none of these have been correlated to Wernicke's syndrome [11–13].

Wernicke's syndrome is the acute phase of thiamine (i.e., vitamin B1) deficiency, and it is characterized by mental confusion, ophthalmoplegia/nystagmus, and ataxia. Then, Korsakoff's syndrome is the chronic phase of thiamine deficiency, and it consists of antegrade and retrograde amnesia, disorientation, confabulation, and limited insight. In addition to damage to the central nervous system, thiamine deficiency can cause peripheral injury (polyneuropathy), and it can have a wide range of severity [14–16].

Prior to the first years of the 2000s [17], Wernicke-Korsakoff syndrome and neuropathy were rarely observed, except mainly in patients who received unbalanced diets or were undergoing rapid weight loss. Only one case was referred to IGB therapy. All such cases were characterized by severe vomiting, which interfered with the intake of food and vitamin supplements. This resulted in severe neurologic manifestations, which mostly affected the lower limbs. All of these patients recovered after vitamin B1 replenishment and without significant sequelae. So, there is a need for a high degree of clinical awareness, with urgent therapeutic intervention required when thiamine deficiency is suspected or is diagnosed.

In more recent years [15], some 50 cases of neurologic symptoms after bariatric procedures have been reported. These cases presented as both peripheral neuropathy (62%) and encephalopathy (31%). Koffman et al. [15] recommended routine monitoring of micronutrient levels and the possibility of neurological complications where there are neurological symptoms after bariatric surgery.

A recent review [4] on Wernicke's syndrome concluded that there is a lack of sufficient data on micronutrient deficiency after restrictive weight-loss interventions (e.g., as for IGB therapy) and that persistent vomiting can be considered as the major determinant of Wernicke's syndrome. This thus leads to impairment of thiamine absorption and, in turn, to thiamine deficiency. However, the duration of such vomiting varies widely among patients, and thus it has not been possible to define a cut-off limit for the onset of Wernicke's syndrome.

Current guidelines do not recommend micronutrient supplementation for all patients undergoing restrictive weight-loss interventions (including for IGB therapy). There is thus a great need for prospective studies to evaluate changes in micronutrients levels under the different types of surgery. Strict monitoring of these patients is needed, because untreated thiamine deficiency can cause serious, and potentially irreversible, neurological damage, or death. Although thiamine deficiency is much more frequent in patients who undergo duodenum and jejunum bypasses (i.e., the preferred absorption sites), patients who undergo IGB therapy are also at an increased risk of thiamine deficiency. During IGB therapy, thiamine deficiency can be the result of restricted nutrient intake, persistent vomiting, and the common noncompliance with vitamin and mineral supplementation.

The treatment of Wernicke's syndrome should begin at the first medical examination, when physicians are responsible for making patients aware that they should not ignore episodes of vomiting (i.e., they should not be accepted, not even to achieve greater weight loss) and aware of the importance of an equilibrated diet. Physicians must also be aware that some eating disorders can appear after bariatric procedures [18]. The patients need to be involved in all of the decisions made, and they must accept the full medically supervised weight-loss program as part of their IGB therapy.

## 4. Conclusions

Although the international literature mainly stresses the possibility of weight regain after IGB therapy [19] and the correlation between weight loss and reduction of obesity-related complications [12], the case presented here shows that IGB therapy must be approached as a multidisciplinary medical program and that patients must be well informed of all of the possible side effects. It is now mandatory to report and carefully investigate any neurological signs that appear during IGB therapy, or after IGB removal. Indeed, the clinical expression of thiamine deficiency (i.e., from neuropathy to encephalopathy) is reversible if diagnosed and treated early.
